# Dendritic spine loss deep in the neocortex and dendrite distortion with diffusion disturbances occur early in experimental pneumococcal meningitis

**DOI:** 10.3389/fnins.2022.912445

**Published:** 2023-01-10

**Authors:** Dario Baronti, Nikola Tomov, Sabrina Hupp, Timothy J. Mitchell, Asparouh I. Iliev

**Affiliations:** ^1^Institute of Anatomy, University of Bern, Bern, Switzerland; ^2^School of Immunity and Infection, College of Medical and Dental Sciences, University of Birmingham, Birmingham, United Kingdom

**Keywords:** *Streptococcus pneumoniae*, meningitis, dendritic spine loss, dendrite diffusion disturbance, Golgi-Cox staining

## Abstract

**Introduction:**

*Streptococcus pneumoniae* (pneumococcus) meningitis is a serious disease with substantial lethality and long-term disability in survivors. Loss of synaptic staining in the superficial layers of the neocortex in rodent models and in humans, and pneumolysin (a major pneumococcal toxin)-dependent dendritic spine collapse in brain slices have been described. It remains unclear how deep in the neocortex more discrete changes are present, how soon after disease onset these changes occur, and whether other properties of dendrites are also affected.

**Methods:**

Using a mouse model of pneumococcal meningitis, we studied changes in the neocortex shortly (3–6 h) after the onset of clinical symptoms *via* modified Golgi-Cox silver staining.

**Results:**

Dendritic changes were present in areas with otherwise unchanged cell numbers and no signs of necrosis or other apparent neuronal pathology. Mature dendritic spines were reduced in the pyramidal neurons running through layers 1–5. Additionally, spine morphology changes (swelling, spine neck distortion), were also observed in the deeper layers 4 and 5 of the neocortex. Immature spines (filopodia) remained unchanged between groups, as well as the dendritic arborization of the analyzed neurons. In a third of the animals with meningitis, massive mechanical distortion of the primary dendrites of most of the pyramidal neurons through layers 1–5 was observed. This distortion was reproduced in acute brain slices after exposure to pneumolysin-containing bacterial lysates (*S. pneumoniae* D39 strain), but not to lysates of pneumolysin-deficient bacteria, which we explain by the tissue remodeling effect of the toxin. Experimental mechanical dendrite distortion in primary neural cultures demonstrated diminished FRAP diffusion of neuronally-expressed enhanced green fluorescent protein (eGFP), indicative of disturbed dendritic diffusion.

**Discussion:**

Our work extends earlier knowledge of synaptic loss in the superficial cortical layers during meningitis to deeper layers. These changes occurred surprisingly early in the course of the disease, substantially limiting the effective therapeutic window. Methodologically, we demonstrate that the dendritic spine collapse readout is a highly reliable and early marker of neural damage in pneumococcal meningitis models, allowing for reduction of the total number of animals used per a group due to much lower variation among animals.

## 1. Introduction

The most common bacteria causing meningitis are *Neisseria meningitidis* and *Streptococcus pneumoniae* (Brouwer and van de Beek, 2018). When *Streptococcus pneumoniae* is involved, one-third of patients do not survive, and half of the survivors remain permanently disabled. Neurological deficits of survivors include changes in behavior, learning disabilities, other severe cognitive deficits, and hearing loss, comprising a heavy socioeconomic burden (Saha et al., 2009). Multiple pathogenic factors contribute to these deficits, including cortical damage, strong neuroinflammation, and secondary pathologic glial cell activation (Mook-Kanamori et al., 2011). The strongest clue for a pathogenic basis of long-term cognitive changes in survivors of meningitis is the synaptic loss in the superficial layers of the neocortex when using PSD95 staining and its correlation with the collapse of dendritic spines in brain slices (Wippel et al., 2013).

Dendritic spines are small protrusions along dendrites that contain the postsynaptic portion of excitatory synapses of the brain. Once mature, PSD95 (postsynaptic density 95) and other postsynaptic scaffold proteins accumulate in the postsynaptic zone, defining areas of excitation. Immunostaining of these proteins can be used for synaptic evaluation (Berry and Nedivi, 2017). Some disadvantages of such immunostaining versus direct spine visualization (e.g., by Golgi impregnation) are variations in tissue staining procedures, despite standardized protocols, as well as the dynamic nature of postsynaptic scaffold markers (Lambert et al., 2017). During visualization, spines with various morphologies can be identified and are generally classified as mature (mushroom, stubby, or thin) or immature and filopodia-like [for an extensive review, see (Berry and Nedivi, 2017)]. In adult animals, approximately 80% of dendritic spines are mature, while 15–20% are immature and filopodia-like. Mature spines are generally more stable over time. They are associated with long-term memory, while immature spines can be reorganized rapidly and are involved in learning and memory (Gipson and Olive, 2017). Dendritic spine loss and synaptic loss accompany various neurological diseases and correlate with cognitive impairment (Herms and Dorostkar, 2016). In some cases, analysis of dendritic spine changes enables a better spatial understanding of the cortex (Mijalkov et al., 2021).

The loss of dendritic spines and synapses in pneumococcal meningitis is attributed to the cholesterol-dependent bacterial cytolysin pneumolysin (PLY), which includes toxin-induced glutamate release from glial cells (Wippel et al., 2013). While these findings clarify the pathogenic mechanisms involved in pneumococcal disability, it remains unclear how early in the course of the disease these effects take place and how deep into the cortex they spread, which is important considering the accumulation of bacteria along the cortical surface. In meningitis tissue samples, PSD95 loss is observed in superficial layers 1–3 after a longer time (36 h to days) of disease development (Wippel et al., 2013). Broader and subtler, but clinically relevant, changes throughout the cortex require different staining approaches and time points of analysis.

The current study analyzes dendritic spine and dendrite changes in an animal meningitis mouse model with the virulent *S. pneumoniae* serotype 2 strain D39, using a modified Golgi impregnation technique and focusing on earlier time points.

## 2. Materials and methods

### 2.1. Animal experiments

All animal experiments were performed in accordance with the Swiss Animal Protection Act (Tierschutzgesetz) and approved by the Cantonal Animal Protection Commission of Canton Bern under license number BE103/20. All animal experimentation methods were performed and reported according to the ARRIVE guidelines^1^. For determination of the number of animals in the experiments, we used the G*Power software (version 3.1.9.6, Franz Faul, University of Kiel, Germany, Supplementary Figure 1) using the following experimental parameters: α error probability 0.05, power 0.95, preliminary control group effect size (spine density in layers I/II) mean ± SD (100 ± 5.6), preliminary meningitis group (spine density in layers I/II) mean ± SD (75 ± 10.66). Calculated effect size was d = 2.94, leading to recommended sample size per an experimental group of 4. We used six in the meningitis group to compensate for probable animal death.

We used 8–12 weeks-old C57Bl/6 mice, randomized into two groups containing equal numbers of males and females. Animals from both groups were treated and subjected to surgery (bacteria-exposed or mock-infected) in parallel, to minimize variation. The animals were obtained from Janvier Labs (Le Genest-Saint-Isle, France), allowed to acclimate for 14 days in an air-conditioned animal facility with a 12/12 dark/light cycle (21 ± 2 ^°^C, 56% relative humidity with lights turned on at 08:00 and turned off at 20:00), and provided with food and water *ad libitum*. Meningitis was induced by injecting 10^4^CFU (colony-forming unit) of D39 bacteria in 15 μl saline in the subarachnoid space using the following coordinates (relative to bregma): mediolateral = midline (0), anteroposterior = 1 mm, and dorsoventral = 4 mm (Chiavolini et al., 2004). The needle was inserted through a skull opening made by a dental microdrill, using a Hamilton microsyringe, controlled by an injection unit gradient step motor with exact flow control (Stoelting Co, Wood Dale, IL, USA) for 6 min. All mock-infected (control) animals received saline-only injection. Animals were anesthetized with 5% isoflurane and maintained with 2% isoflurane (Baxter International, Unterschleißheim, Germany). Before incision, the skin was infiltrated with a lidocaine/bupivacaine mix (1:1 total volume of 0.2 ml/mouse, max 4 mg/kg total; xylocaine 1%, carbostesin 0.25%, obtained through the veterinary university pharmacy). Paracetamol (3.5 mg/ml, Dafalgan Syrup, Upsa, Zug, Switzerland) was provided in post-surgery drinking water throughout the experiment. Animals were checked following a scoresheet at 6, 12, 15, and 18 h using the following parameters: activity, body temperature, coat, posture, grimace, body condition, and neurological status. Status score of animals was calculated as a sum as follows: activity (0—normal, 1—increased/decreased/mildly diminished, 2—diminished, 3—severely diminished, 4—coma), coat (0—normal, 1—diminished grooming, 2—piloerection), posture (0—normal, 1—slightly hunched back, 2—severely hunched back), grimace scale (0—open eyes and straight ears, 1—orbital tightening, cheek bulge, 2—orbital tightening, cheek bulge, and flat ears), adapted body condition score index (Ullman-Culleré and Foltz, 1999) (0—BCS3, 2—BCS2, 3—BCS1), neurologic exam (0—normal, 1—ataxia, 2—limb paresis/paralysis/epileptic seizures, 3—status epilepticus). Animals were euthanized when individual score items reached two or the total score reached six.

### 2.2. Neuronal and slice cultures, treatments

Primary mouse neuronal cultures were prepared from the cortices of day 16 embryos of C57BL/6JRj mice. Pregnant mice were euthanized following legally approved regulations. Uteri were removed, embryos extracted, and cortices separated from the rest of the tissue, with meninges removed beforehand. Briefly, the tissue was dissociated into cell suspensions and plated in eight-well coverslip bottom dishes (Sarstedt AG, Nuembrecht, Germany) coated with poly-L-ornithine (PLO, Sigma–Aldrich). The growth medium, BME, was supplemented with 1% fetal calf serum (FCS, PAN Biotech, Aidenbach, Germany), 1% penicillin/streptomycin, 2% B-27, and 1% glucose (all from Thermo Fisher). At day 3 after seeding, the neurons were transduced with synapsin-eGFP adenovirus (AAV-hSYN1-mSYP/eGFP-WPRE) (Vector BioLabs, Malvern, USA) for diffuse neuron-specific EGFP expression in the cytosol of neurons. Ten to fourteen days after preparation, cells were ready for use.

Acute slices were prepared from the brains of newborn C57Bl/6JRj mice at postnatal day (PD) 10–12, according to a previously published protocol (Hupp et al., 2021). Under isoflurane anesthesia, decapitation, skull opening, and cranial plate removal were performed, and the brain was transferred to PBS. The cerebellum was cut off and discarded, and the cerebrum glued cut-surface down to a specimen holder with Vetbond (3M Company, Saint Paul, MN, USA). A total of 340 μm-thick sections were prepared on a Leica VT1000 S sectioning vibratome (Leica Microsystems GmbH, Wetzlar, Germany). Coronal sectioning was carried out at the level of the sensorimotor cortex, using Plates 19–21 of the “*The mouse brain in stereotaxic coordinates”* (Paxinos and Franklin, 2001) as reference. During slicing, brains were kept in constantly oxygenated (95% O_2_/5% CO_2_ mix, Carbagas Depot F. + H. Engel, Bern, Switzerland), room temperature artificial cerebrospinal fluid (aCSF) containing glucose (12.5 mM) and 1% penicillin/streptomycin [all from Gibco, Thermo Fisher Scientific (Switzerland) AG, Basel, Switzerland]. For storage of up to 30 min, freshly cut slices were kept in oxygenated aCSF at room temperature in cell strainers (100 μm pore size, Corning, Inc., Corning, NY, USA). Slicing and storage were performed at room temperature rather than on ice, due to our experience that cooling causes rapid and pronounced shrinkage of dendritic spines. For recovery, the slices in cell strainers were kept for 30 min at 33^°^C in aCSF in a six-well plate (under constant oxygenation) before temperature was increased to 37^°^C for a subsequent treatment period of 5–12 h (also under constant oxygenation). The slices were incubated with lysates from D39 pneumococci and D39 pneumococci without PLY expression (D39 ΔPLY). Bacterial lysate amounts in all experiments were equivalent to 7 × 10^7^ CFU/ml (within the range of bacterial density in the CSF) (Feldman, 1976).

Artificial cerebrospinal fluid was prepared using the following components (all from Carl Roth GmbH + Co. KG, Karlsruhe, Germany unless otherwise specified): 119 mM NaCl, 2.5 mM KCl, 1.2 mM NaH_2_PO_4_, 25 mM NaHCO_3_, 12.5 mM glucose, 2 mM MgSO_4_, 2 mM CaCl_2_, 1% penicillin/streptomycin (ThermoFisher). Once carbogenated, the pH adjusted to 7.3–7.4 due to the buffer capacity of the solution.

### 2.3. Bacterial cultures

The *S. pneumoniae* bacterial strains [the wild-type D39 strain (D39) and the D39 PLY-deficient mutant strain (ΔPLY)] were generously donated by Jeremy Brown (University College London, UK). The different strains were plated on blood agar plates (Columbia agar with 5% sheep blood; Oxoid Limited, Hampshire, UK) and incubated at 37^°^C under anaerobic conditions overnight. Several colonies were picked and grown to mid/late log phase (OD_600_ of 0.7) in brain heart infusion broth (BHIB; Becton Dickinson and Company, Le Pont de Claix, France). Cultures were then centrifuged and washed three times with phosphate-buffered saline (1x PBS), and serial dilutions were plated on blood agar plates according to the Miles and Misra method (Miles et al., 1938) to determine the colony-forming units (CFU) per milliliter.

Following euthanasia, half of the cerebellum, 50 μl of the blood, and the spleen were each isolated and homogenized in 1 ml sterile PBS using a RW16 mechanical tissue homogenizer (IKA Werke AG, Germany). Serial dilutions on blood agar plates were performed to confirm the presence of infection and to determine the number of pneumococci.

### 2.4. Tissue processing and staining

Brain was removed and fixed in 1.5% formaldehyde in PBS immediately after euthanasia for 4 h and subsequently washed in PBS. Coronal sections with a thickness of 100 μm, allowing for confocal imaging, were obtained by sectioning using a Leica VT1000 S vibratome (Leica Microsystems GmbH, Wetzlar, Germany). Sections between 1 and 0.5 mm anterior to bregma (Paxinos and Franklin, 2001) were used for impregnation. Brain slice-modified Golgi silver impregnation was performed using the SliceGolgi Kit (Bioenno Tech LLC, Santa Ana, CA, USA) according to manufacturer instructions, with empirically determined optimal constant incubation durations, so the staining intensity was uniform through the batches. The impregnated sections were mounted on gelatin-coated slides, air-dried, cleared in xylene, and mounted with Entellan (Carl Roth, Karlsruhe, Germany). For DAPI (4’,6-diamidino-2-phenylindole; Sigma) staining, sections were briefly incubated in ice-cold methanol for membrane removal, followed by 1 h incubation with the stain at 1 μg/ml in PBS.

Following treatment, the acute brain slices from juvenile mice (see preparation above) were fixed with 1.5% formaldehyde for 2 h. After washing with PBS, slices were stained with NeuroTraceTM DiI stain (Thermo Fisher) by direct application of the dye onto the cortical region of the slices with a very fine Tungsten needle (Fine Science Tools, North Vancouver, Canada), prepared as described hereafter. Several microliters of a 2% solution of DiI crystals in ethanol were placed on a piece of Parafilm, then the tungsten needle was carefully dipped into this solution and left to dry, allowing small DiI crystals to form on its surface. DiI crystals were subsequently inserted into the cortex by punching them through the cut surface into neocortical layers 3, 4, and 5. Slices were left for 24 h for dye diffusion, briefly postfixed, washed, and mounted in Mowiol (aqueous).

Identification of neocortical layers in silver impregnation tissue was performed following the classical definitions set by K. Brodmann and with the help of the Allen Brain Atlas^2^ (Wang et al., 2020). We focused our analysis on the primary and secondary somatosensory cortex (S1-S2) in the stereotactic space between 0.5 and 1 mm ventrally from bregma (Paxinos and Franklin, 2001), which is also 1.2–1.3 mm behind the injection point (Supplementary Figure 2). The pyramidal motoneurons (and the secondary branches of their apical dendrites) were our main cells of interest, both due to their abundance and distinctive morphology, and due to them being strongly affected by treatment (Supplementary Figure 3).

### 2.5. Histology imaging and dendritic spine analysis

Fluorescence images were acquired on a Zeiss LSM 880 with Airyscan confocal system using 63x oil immersion objectives (Carl Zeiss AG, Oberkochen, Germany) and either green (488 nm) or red excitation wavelengths (561 nm) at 2,048 × 2,048 pixel resolution as Z-stacks. Silver-stained sections were imaged on the same system using laser illumination and a photomultiplier transmission imaging channel, allowing high-resolution light microscopy images (Supplementary Figure 4). For DiI staining of acute brain slices, the same microscopy system was used with an excitatory laser line of 561 nm and a 63x objective. Image analysis and reconstruction were performed using ImageJ (ver. 1.52p; NIH, Bethesda, USA) with MBF “ImageJ for Microscopy” Collection from Tony Collins and ImageJ (with Fiji add-on package, Johannes Schindelin and team). For clarity of the image during analysis, the Z-stacks were projected in one plane. Classification of the spines was performed according to established criteria (see Figure 3B; Bosch and Hayashi, 2011; Pchitskaya and Bezprozvanny, 2020). Only dendritic spines on the secondary branches of the apical dendrite of large pyramidal neurons (Supplementary Figure 3) were analyzed out of practical reasons of identifiability. Per animal and layer set (I–III, IV–V, and VI), we analyzed 5–10 neurons. Dendrite arborization was assessed by the Sholl Analysis plug-in of ImageJ, using starting radius of 10 μm, ending radius—130 μm and radius step size of 5 μm. The analysis of all sections/staining was performed by a double-blinded procedure, blinding both samples during staining, during imaging and during evaluation—the latter performed by Rename Expert Software (Gillmeister Software, Bad Harzburg, Germany) (Figure 3A).

**FIGURE 1:**
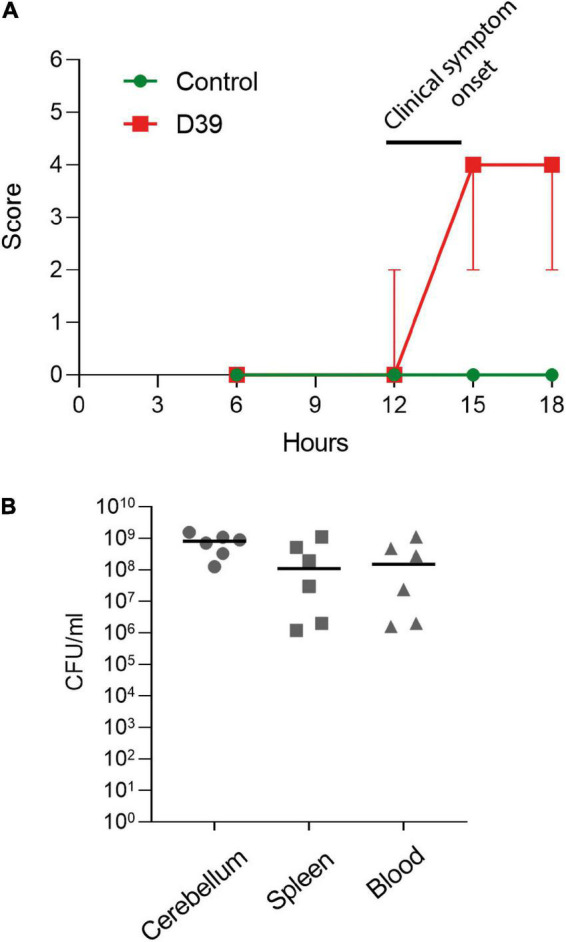
Characterization of the subarachnoid injection mouse meningitis model. **(A)** Clinical score of the animals (see section “2. Materials and methods”). Values represent median ± 95% CI. **(B)** Determination of the colony-forming unit numbers of *S. pneumoniae* samples from the cerebellum, spleen, and blood of D39-infected mice. Each animal is indicated as a separate symbol in the scatter plot. For mock-infected animals (control), *n* = 4 animals, for D39-infected mice (D39), *n* = 6.

**FIGURE 2:**
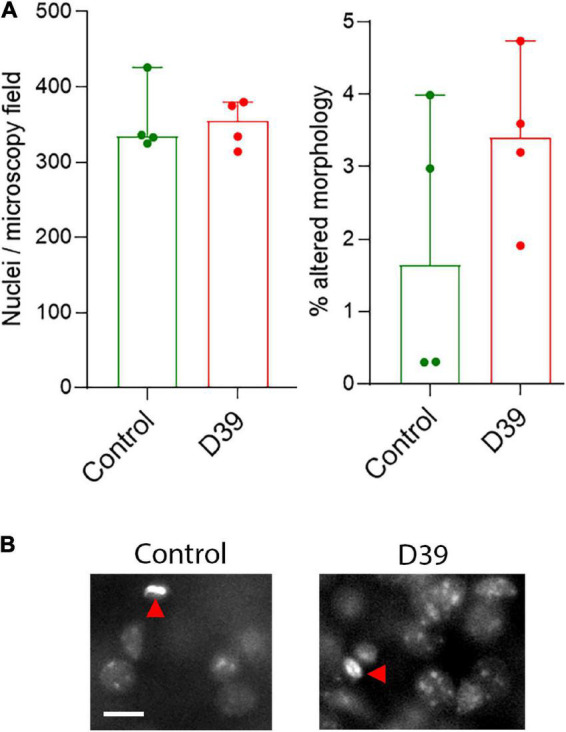
**(A)** Lack of significant difference in the number of nuclei in layers 1–3 (left) and in the fraction of nuclei with pathologic (condensed, fragmented) morphology in both animal groups. All values represent median ± 95% CI, Mann-Whitney U-test. Each animal is indicated as a separate symbol in the scatter plot. For mock-infected animals (control), *n* = 4 animals, for D39-infected mice (D39), *n* = 4. **(B)** Morphological example of nuclear stain of cells in the superficial layers of the neocortex. We did not observe any polymorphonuclear lymphocytes (neutrophils) to indicate an increased parenchymal infiltration at this point. Red arrows point to individual cells with altered (in this example pyknotic) nuclear morphology. Scale bar: 10 μm.

**FIGURE 3:**
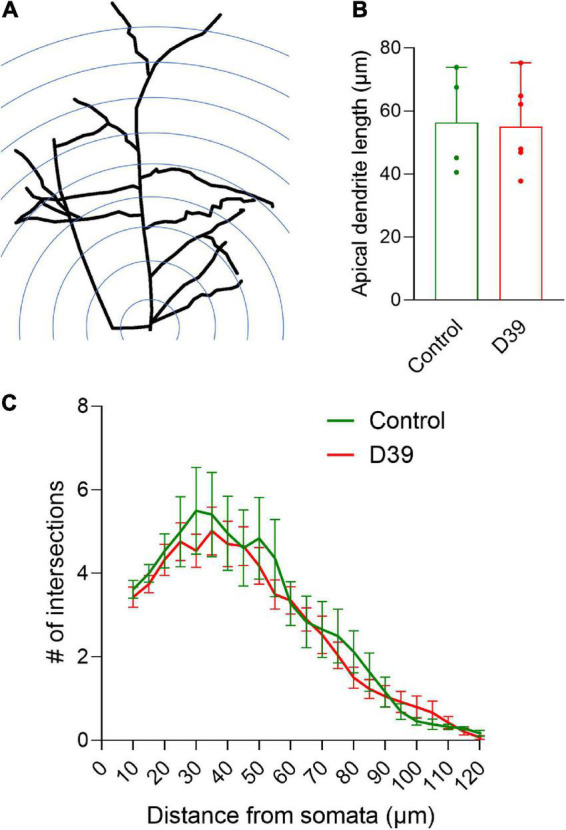
Dendritic arborization of pyramidal neurons in layers I–III. **(A)** Diagram of the Sholl analysis method for dendritic intersection quantification (for details see section “2. Materials and methods”). **(B)** Average length of the apical dendrite (in both groups, there were equivalent portions of pyramidal neurons with much shorter apical dendrites, presumably staining artifact). Values represent median ± 95% CI. **(C)** Distribution of the dendritic intersections between both groups demonstrates identical shapes. Measurement radius was limited to 130 μm to minimize the effect of staining artifacts along longer neurites. Values represent mean ± SEM.

### 2.6. Live-cell imaging of distorted neurites

After 10–14 days following transduction, live-cell imaging was performed on a Zeiss LSM 880 using 63x oil immersion objectives (Carl Zeiss AG, Oberkochen, Germany), using green laser (488 nm) with minimal power of 0.5–1% and photomultiplier power at 800–820 V. The GFP-transduced neurons were maintained in HEPES-buffered imaging buffer containing 142 mM NaCl, 5.4 mM KCl, 1.8 mM CaCl_2_, 1 mM NaH_2_PO_4_, 25 mM HEPES, 5 mM glucose, and 1 mM MgSO_4_, pH = 7.4. During visualization, using a custom-built micromanipulator attached to a gel-loading plastic pipette tip (Sarstedt, Germany), mechanical distortion of the dendrites was performed by bending their axis beyond 90^°^ and analyzed. We used FRAP (fluorescent recovery after photobleaching) to test the diffusion of fluorescent EGFP in mechanically intact and mechanically distorted dendrites (Zheng et al., 2011; Xia et al., 2016). Selected dendrite segments were bleached immediately before and after mechanical distortion for analyzing the free diffusion of monomeric EGFP molecules [laser power of 100% (561 nm), 10 rounds of bleaching] and followed for 60 s thereafter.

### 2.7. Statistics

Statistical analysis was performed using GraphPad Prism 9.1.0 for Windows (GraphPad Software Inc., La Jolla, CA, USA). For comparison of two groups differing in one parameter, the Mann–Whitney test was applied. For comparison of more than two groups, one-way ANOVA on ranks (Kruskal-Wallis H-test) with Dunn post-test were performed. For all analyses, non-parametric tests were performed, recommended both for smaller samples (<10 values) or for higher number samples independent on their normality (Motulsky, 1995). For the analysis of FRAP curves, one-phase exponential regression was applied. The results show the mean value and standard error of the mean unless indicated otherwise. For all experiments, a significance level (α) for rejection of the null hypothesis above 0.05 was defined, thus considering significant *p*-values below 0.05. In all scatter plots, each single sample (animals for tissue experiments, neurons in cell culture experiments) is indicted by a symbol, the exact number (*n*) is added to the figure legends. In each graph, the exact value of *p* is presented (if below 0.05).

## 3. Results

In the *in vivo* model of pneumococcal meningitis, first mild symptoms were observed at 12 h after the inoculation of bacteria. Six hours later, the meningitis animals demonstrated diminished activity and an increase in their pathologic score (Figure 1A). Analysis of the bacterial numbers confirmed meningitis and the beginning of blood dissemination (Figure 1B)—pneumococci in significant numbers were already present in the bloodstream and in the spleen.

Using modified Golgi silver impregnation on cortical sections of fixed brains, we visualized the dendritic trees of the neurons in the neocortex along the whole curvature (see section “2 Materials and methods”). In some samples, there were areas of nearly missing staining, but analysis of adjacent sections confirmed it is a staining artifact. No loss of fine chromatin structure, shrinkage, or fragmentation of the nuclei was observed throughout the cortex (Figures 2A, B), suggesting no direct cytolytic effect of bacterial products.

**FIGURE 4:**
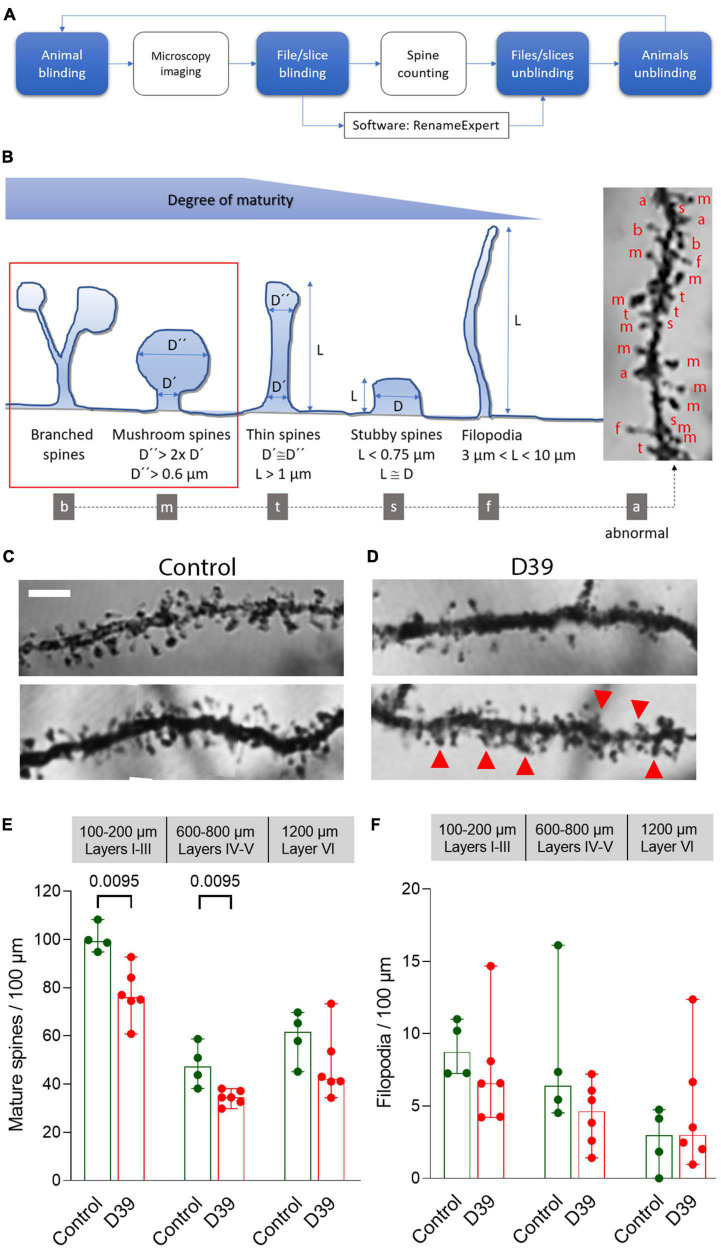
Dendritic spine loss and pathologic spine morphology in meningitis animals. **(A)** Schematic workflow diagram of the evaluation of dendritic spines with two steps of sample blinding to reduce subjectivity. **(B)** Major dendritic spine morphologies and criteria we used for classification of mature and immature spines (see section “2. Materials and methods”) with examples of different types of spine identification (on a silver impregnation tissue sample right). **(C)** Normal morphology of the spines on secondary branches of the apical dendrite of pyramidal neurons in layer 4. Most spines are mature, exhibiting a mushroom form with a well-defined head and neck; dendrites demonstrate regular morphology with parallel borders. Scale bar: 5 μm. **(D)** Eighteen hours after initiation of meningitis and 12–15 h after the onset of symptoms, dendrites demonstrate reduced numbers of spines in the somatosensory cortex (S1-S2). Abnormal protrusions/not identifiable structures are indicated with red arrows. All images represent a single layer overlay of the stacks, used for spine analysis (see Supplementary Figure 4). **(E)** Significantly reduced numbers of mature dendritic spines with normal morphology through layers 1–5. **(F)** Filopodia, representing immature spines, remain unchanged through the cortex in the meningitis animals versus healthy controls. All values represent median ± 95% CI, Mann-Whitney U-test, exact *p*-value is presented on the graph (if below 0.05). The number of animals in each group is indicated by the symbol points in the scatter plot [for mock-infected animals (control), *n* = 4 animals, for D39-infected mice (D39), *n* = 6], each group contains equal numbers of male and female animals.

Next, we focused on the dendritic structures of the pyramidal neurons in various layers of the neocortex (specifically the somatosensory cortex S1-S2). Pyramidal neurons in layer I–III of the meningitis animals demonstrated proper arborization of the dendritic tree excluding acute toxicity (Figures 3A, C). Apical dendrite length was also identical between groups (Figure 3B). Next, we analyzed dendritic spine morphology of pyramidal neurons, following a double-blinding (animal and image blinding) protocol with human evaluation (see section “2 Materials and methods” and Figure 4A). Unblinding followed the end of the evaluation. We counted mature spines as major morphological substrates of established synapses (Lippman and Dunaevsky, 2005) (see section “2 Materials and methods” and Figure 4B for evaluation criteria and examples). Mature dendritic spine loss was apparent in neocortical layers 1–3 (Figures 4C–E; *p* = 0.0095, Mann-Whitney U-test), reaching layers 4 and 5 (600–800 μm from the cortical surface; Figures 3C–E; *p* = 0.0095, Mann-Whitney U-test). Control animals showed normal spine configuration, with the majority demonstrating mature morphology, being either mushroom-shaped or branched (Figure 4C). In meningitis animals, there was a general loss of mature spines and presence of areas with abnormal, swollen spines, spines with aberrant form (brush-like appearance), and spines with bent necks throughout neocortical layers 1–5 (Figure 4B, “a”; Figure 4D, Red arrows). Filopodia, representing immature spines, remained unchanged through the cortex in the meningitis animals versus healthy controls (Figure 4F).

**FIGURE 5:**
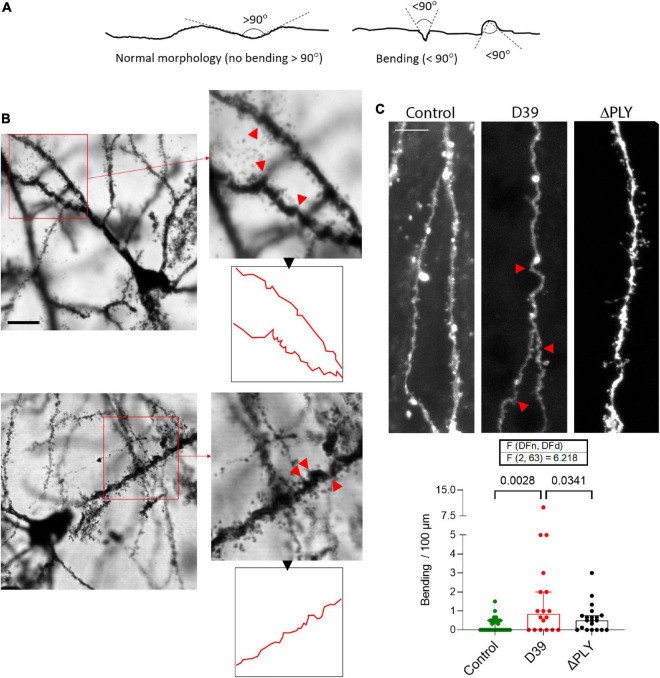
Mechanic distortion of apical dendrites. **(A)** Morphological criteria for determination of the bending in dendrites. Any fragment of the dendrites with neighboring elements at an angle of 90^°^ or lower is considered bended. **(B)** Example of two neurons (layer 4) from two animals, representative of the multiple neurons in their neocortex with massive mechanical twisting/bending. Red arrows indicate the sharply bended/distorted fragments of the dendrites, outline in red demonstrates the neurite contours. Scale bar: 10 μm. **(C)** Analysis of the number of distorted points (<90^°^ bending) per 100 μm of DiI-strained primary apical dendrites of cortical pyramidal neurons in acute brain slices treated for 6 h with bacterial lysates originating from wild-type D39 pneumococci (D39) or from D39 pneumococci with knocked-out PLY gene (ΔPLY). All values represent median ± 95% CI, One-way ANOVA, exact *p*-value is presented on the graph (if below 0.05). The number of analyzed neurons in each group is indicated by the symbol points in the graph (mock-treated (control), *n* = 28 neurons; D39, *n* = 18 neurons; ΔPLY, *n* = 20 neurons), on average 1–2 neurons per acute slice.

In a third of the animals with meningitis, multiple neurons with substantial distortion and apical dendrite bending were observed throughout the neocortex (Figure 5). Dendrite bending was defined as bending of any fragment of the dendrites with neighboring elements at an angle of <90^°^ (Figure 5A). The changes in the third of the meningitis animals (2 of 6 animals) was apparent, wide-spread and characteristic (Figure 5B). We observed the similar effect in earlier experiments in acute mouse infant brain slices with continuous oxygenation and exposure to D39 lysates for 6 h (Figure 5C), which demonstrated significantly increased numbers of bendings versus the mock-treated control (*p* = 0.0028, One-way ANOVA). All analyses of dendrite bending were performed on the primary apical dendrite (Supplementary Figure 2). At the same time, brain slices exposed to D39 ΔPLY lysates demonstrated significantly less bendings versus the D39-lysate-treated slices (Figure 5C; *p* = 0.0341, One-way ANOVA). As described before (Wippel et al., 2013), dendritic spine loss was present when PLY was present, and it was eliminated in PLY knockout strains. The fact that dendrite bending was not observed in any of the mock-infected animals (or in mock-treated slices), eliminates a possible effect of tissue distortion during Golgi staining procedure.

**FIGURE 6:**
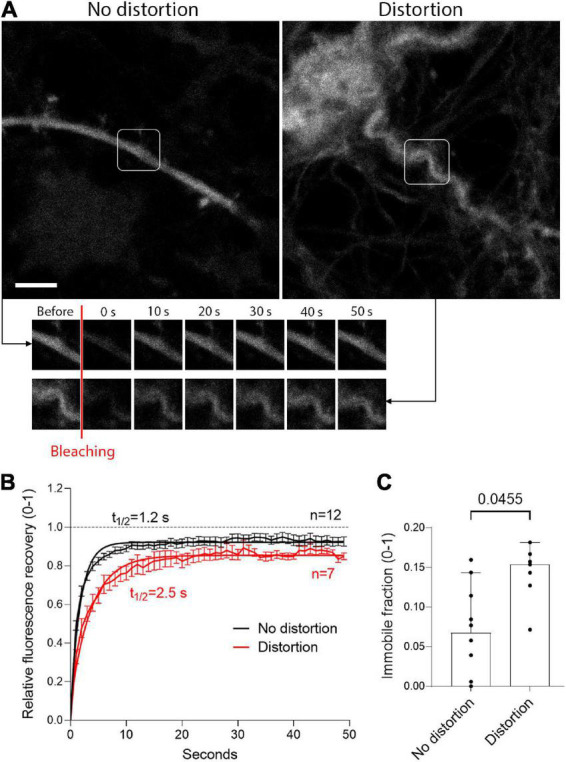
Delayed diffusion in mechanically distorted fragments of dendrites. **(A)** Enhanced green fluorescent protein (eGFP) expression under the control of the neuron-specific synapsin promoter in neurons without mechanical distortion (left) and after distortion (right), with images at several time points after bleaching. Scale bar: 5 μm. **(B)** Fluorescent recovery after photobleaching (FRAP) with one-phase exponential regression fitting demonstrates doubled recovery halftime of EGFP after mechanical deformation (values represent mean ± SEM). **(C)** Significant increase in the immobile fraction at the plateau of the FRAP curve. All values represent median ± 95% CI, Mann-Whitney U-test, exact *p*-value is presented on the graph (if below 0.05). The number of analyzed neurons in each group is indicated by the symbol points in the graph (for the non-distorted group, *n* = 12 neurons; for the distorted group, *n* = 7 neurons).

To analyze the pathogenic and functional implications of dendrite deformation, we used FRAP to test the diffusion of fluorescent EGFP (following expression of EGFP under the control of synapsin promoter by AAV in primary neurons, see section “2 Materials and methods”) in mechanically intact and distorted (mechanically) dendrites (Figure 6A). In FRAP, strong laser light is used to destroy the fluorescent molecules in a small volume of a cell, as the fluorescent recovery due to transport or diffusion of non-destroyed fluorescent molecules from the vicinity into the “dark” area is measured. In mechanically deformed dendrites, at the place of deformation, the diffusion of EGFP was reduced 2-fold (t_1/2_ = 1.2 s for non-distorted versus t_1/2_ = 2.5 s for distorted dendrites; Figure 6B), and the fraction of immobile molecules in the fragment of deformation was increased 2-fold (Figure 6C; *p* = 0.0455, Mann-Whitney U-test).

## 4. Discussion

Our results demonstrate that in pneumococcal meningitis, cortical tissue is affected by the loss of dendritic spines more extensively and much earlier in the disease course than previously thought. We studied a clinically relevant and diagnostically realistic early time point - the time window of 3–6 h after the onset of clinical symptoms corresponds to a very timely clinical diagnosis, which is often not the case, especially when access to medical care is limited. Pathologic dendritic spine alterations extended to 800 μm under the surface of the neocortex within hours after disease onset. We also observed, for the first time, mechanical changes in the configuration of dendrites and we confirm in culture that such distortion can hamper the diffusion of molecules.

Dendritic spine loss in acute brain slices after PLY challenge and the loss of PSD95 staining in the superficial brain cortical tissue (layers 1–3) after pneumococcal meningitis in mice (36 h after disease onset) and in humans (various times after disease onset) have been described previously (Wippel et al., 2013). Until now, the status of the dendritic spines, as well as the involvement of deeper layers, has remained unclear. The previous works do not answer several important questions, such as (i) whether deeper neocortical changes, not detectable through PSD95 staining, are present; (ii) whether these effects occur early or late in the disease course; (iii) whether subtler changes not immediately detectable in thin sections are present; (iv) what the status of the neurite tree as a whole in the cortex is (i.e., is the effect affecting dendrites as well as spines); and (v) whether the findings in the cortex of animal brains with meningitis reflect the same phenomena observed in acute brain slices, which, although well-accepted as a model, still differ from intact brains in terms of pathogenic factor accessibility. The current study answers all these questions. The spine loss affected only mature spines, but not filopodia. The latter represent immature, not established dynamic spines (Berry and Nedivi, 2017). They do not host synapses and established receptor densities contrary to mature spines. This, combined with the presence of NMDA-mediated synaptic damage in pneumococcal meningitis (Wippel et al., 2013), may explain why only mature spines were affected. This is of importance for the preservation of the neural tree ability to regenerate synapses once the infection is resolved.

The current work demonstrated a major new finding that has remained underestimated in earlier works due to the specificity of DiI staining—namely, the presence of dendrite distortions. DiI staining, which can be assessed only in intact neurons [damaged neurons are not stained—the dye diffuses only along intact membranes and does not cross to other cells (Liu et al., 2008)], biases the analysis solely to normal neurons.

The physiology and morphology of neurons are unique among all other cell types in the body. Their large size requires fine coordination between distant compartments. It is not uncommon in other diseases to observe initial degeneration of distant compartments before degeneration of the neural body. Most studies investigating processes of neuronal damage have focused on axonal damage (such as in multiple sclerosis, meningitis, and many other conditions) (Coleman and Perry, 2002; Nau et al., 2004). Axons are particularly vulnerable to transport disturbances due to the proportionally higher need for active transport, their small diameter, and (often) substantial length. While dendrites are shorter and wider than axons, they rely more on passive diffusion than active transport (Overly et al., 1996; Lardong et al., 2009). The dendrite changes observed both in one-third of the animals and in D39 lysate-exposed slices, was an interesting finding that can be easily neglected in histological samples. The presence of dendrite alterations in acute slices with identical patterns only after exposure to bacterial products was also informative, indicating that this finding was not an artifact of the Golgi staining procedure, which is generally known to induce tissue distortion. Furthermore, the distortion was not observed in the control animals, in which the staining procedure was identical. Analysis of dendrite distortion in brain slices using knockout bacteria indicated a role of PLY. PLY massively reorganizes the cytoskeleton of glial cells through microtubule and actin remodeling, leading to morphological cellular and tissue changes (Iliev et al., 2007, 2009; Hupp et al., 2012). We suggest that similar mechanisms contribute to the mechanical deformation of dendrites, leading to irregular distribution of tension within the tissue and subsequent conformational changes in neurites. Last, but not least, the lack of nuclear alterations and the missing changes of dendritic arborization did not reveal increased cytotoxicity caused by the bacterial products, indicating that the neuronal damage started primarily from the spines.

Dendrites are primarily dependent on passive diffusion of molecules (Overly et al., 1996; Lardong et al., 2009). Dendrite transport is primarily based on passive diffusion, although some active transport also occurs (Overly et al., 1996). Local synthesis complements transport due to the need to overcome longer distances, especially in the neocortex, where dendrites can reach significant lengths (Eberwine et al., 2001). Dendrite degeneration in the presence of intracellular aggregates (in Alzheimer’s disease, Huntington’s disease, and others), extracellular toxic factors, and metabolic challenges is known, and disturbances of dendritic transport are suggested to play a role in these processes (Park et al., 1996; Scheff and Price, 2003; Lee et al., 2011; Kelley et al., 2019; Chung et al., 2020). The removal of PLY eliminated the dendritic bending effect, confirming the role of cytolysin in it, and the sensitivity of dendrites to the toxin (Figure 4B). Our findings link the observed morphological alterations and the subsequent functional change—molecular diffusion disturbance. Whether this disturbance has a strong effect on dendrite function and the observed spine loss remains unclear. We failed to observe predominant spine loss in the vicinity/distally from the distorted fragments of the dendrites, but this may be due to the relatively short experimental duration. How these dendritic changes would affect longer-term effects of the disease remains to be clarified. We believe, however, that dendritic distortion may have implications for other disease conditions with substantial levels of mechanical tissue effects, such as traumatic brain injury, acute brain swelling, and hematoma formation during bleeding, among others.

Correlating the findings from three experiment series, mechanical changes hint toward structural alterations in the biophysical properties of the nervous tissue, exposed to bacterial toxin. Since PLY causes massive cytoskeletal and shape changes in glial cells (Iliev et al., 2007; Förtsch et al., 2011), we believe they are the major factor of dendrite distortion in the tissue. Furthermore, we have previously demonstrated, that PLY reduces extracellular matrix density (Hupp et al., 2012). No deformation of dendrites by lysates or toxin in primary neural culture was expected or observed, presumably due to the lack of extracellular matrix or glial cells. The exact mechanism behind the present findings remains way beyond the scope of this study. While we mimicked the distortion of the dendrites in culture mechanically, we cannot be completely confident that in tissue this deformation is only mechanical and that no other complementary factors (e.g., inflammatory) play a role.

Patients with bacterial meningitis often receive medical treatment once symptoms are present and the disease is clinically advanced. We analyzed the tissues 3–6 h after the onset of obvious clinical symptoms, which corresponds to a very timely window of clinical diagnosis in humans, which, unfortunately, cannot always be achieved. Because of this, it remains important to know which brain tissue changes are reversible (e.g., swelling), partially reversible (dendritic spine loss), or irreversible (permanent loss of cells). The loss of dendritic spines can be partially compensated later if neuroprotective and anti-inflammatory therapies start as early as possible. Specific regenerative treatments [such as brain-derived neurotrophic factor (BDNF)], especially promoting synaptogenesis can be very promising. On a larger scale, our data underscore once again the need of proper disease surveillance on a public health level to identify outbreaks, facilitate diagnosis, protect vulnerable groups and if possible—perform preventive immunizations.

A critical translational aspect of our work is that the thickness of the mouse neocortex in the sensorimotor area—approx. A total of 1 mm—is lower than that of humans, which ranges from 2 to 4.5 mm (Fischl and Dale, 2000). The depth of the effects in the mouse neocortex was 600–800 μm within the time window of our analysis, corresponding to the thickness of human layers 1–3 (depending on the exact region of the brain) and, in some cases, layer 4 (DeFelipe et al., 2002). Synaptic density in the superficial layers of mammalian brains is especially high (Sherwood et al., 2020), and most of the damaging effects are present in both mouse and human samples (Wippel et al., 2013). Even if human tissue effects were limited to 1 mm in depth, this still represents a physiologically significant portion of the neocortex with plenty of synapses.

Earlier works that analyzed the effect of pneumococci and their factors on dendritic spines relied on an acute brain slice system using slices dipped in artificial cerebrospinal fluid containing lysates or bacteria (Hupp et al., 2021). While such a system allows multiple treatments in parallel and a reduction in the number of animals used, it presents a major drawback—toxic factors access the tissue from all sides, improving toxic factor penetration. This does not precisely correspond to the natural penetration of pathogenic factors through the surface of the neocortex in meningitis. Thus, the finding that bacteria and their toxic factors impose similar effects on dendritic trees in intact brains as in acute slices is key, confirming the pathologic relevance of the acute slices as a valid experimental system for meningitis studies. Methodologically, on animal modeling level, the proposed dendritic spine readout in a subarachnoid injection system provides much lower variations among animals, improving the readout in a much lower number of animals than in equivalent setups, where cell death, necrosis or other markers of damage are used (Leib et al., 2000; Bottcher et al., 2004; Spreer et al., 2006). Therefore, we believe that the present study has also translational 3R (“reduce, refine, replace,” regarding animal experiments) importance, helping avoiding unnecessary animal use (Flecknell, 2002).

Understanding the level and type of damage in the neocortex during meningitis at the synaptic level is key to successful meningitis therapy and the elimination of subsequent effects. The next level of analysis should focus on real-time functional tissue visualization and analysis to precisely plan the most effective time window of treatment and therapy approach.

## Data availability statement

The datasets presented in this study can be found in online repositories. The names of the repository/repositories and accession number(s) can be found below: https://doi.org/10.48620/39.

## Ethics statement

This animal study was reviewed and approved by Cantonal Animal Protection Commission of Canton Bern under license number: BE103/20.

## Author contributions

DB, NT, SH, and AI performed the experiments. TM provided the materials. AI and NT analyzed the data. All authors contributed to manuscript writing and approved the submitted version.

## Footnotes

1
https://arriveguidelines.org

2
https://mouse.brain-map.org/static/atlas
