# Supplementary figures and images for: Dendritic spine loss deep in the neocortex and dendrite distortion with diffusion disturbances occur early in experimental pneumococcal meningitis
Source: Front Neurosci. 2023 Jan 10;16:912445. doi: 10.3389/fnins.2022.912445 (PMC9871924; doi:10.3389/fnins.2022.912445)

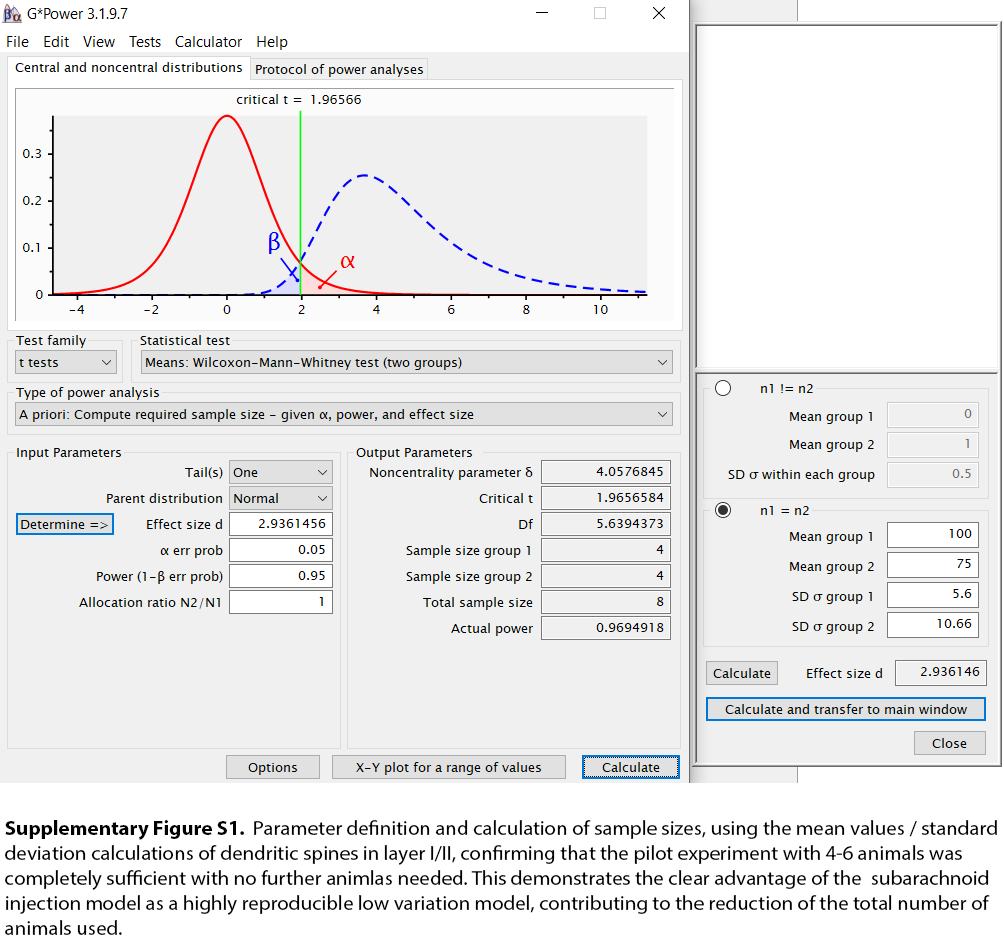

Supplement: Supplementary file 1 [file Image_1.TIF]

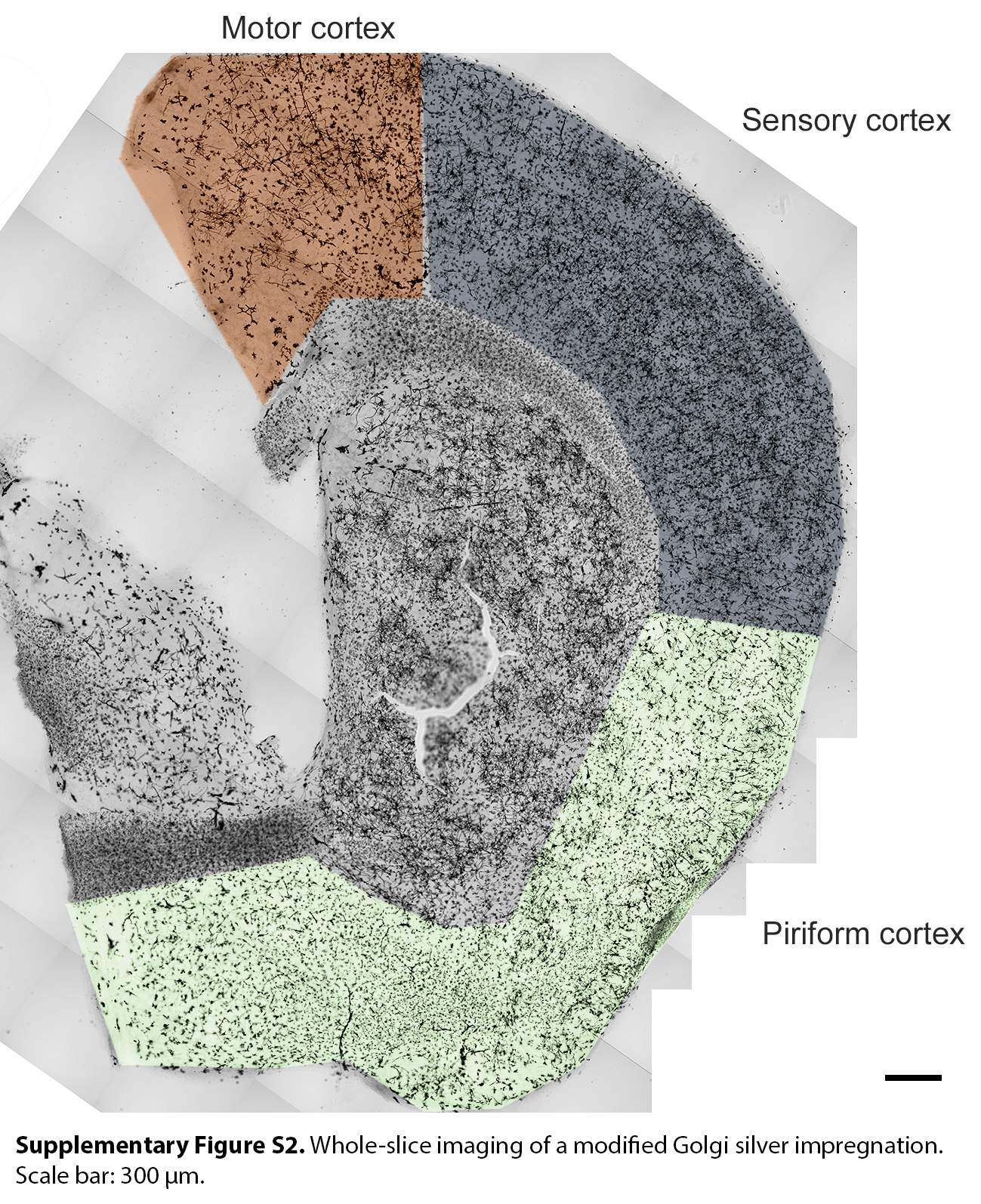

Supplement: Supplementary file 2 [file Image_2.TIF]

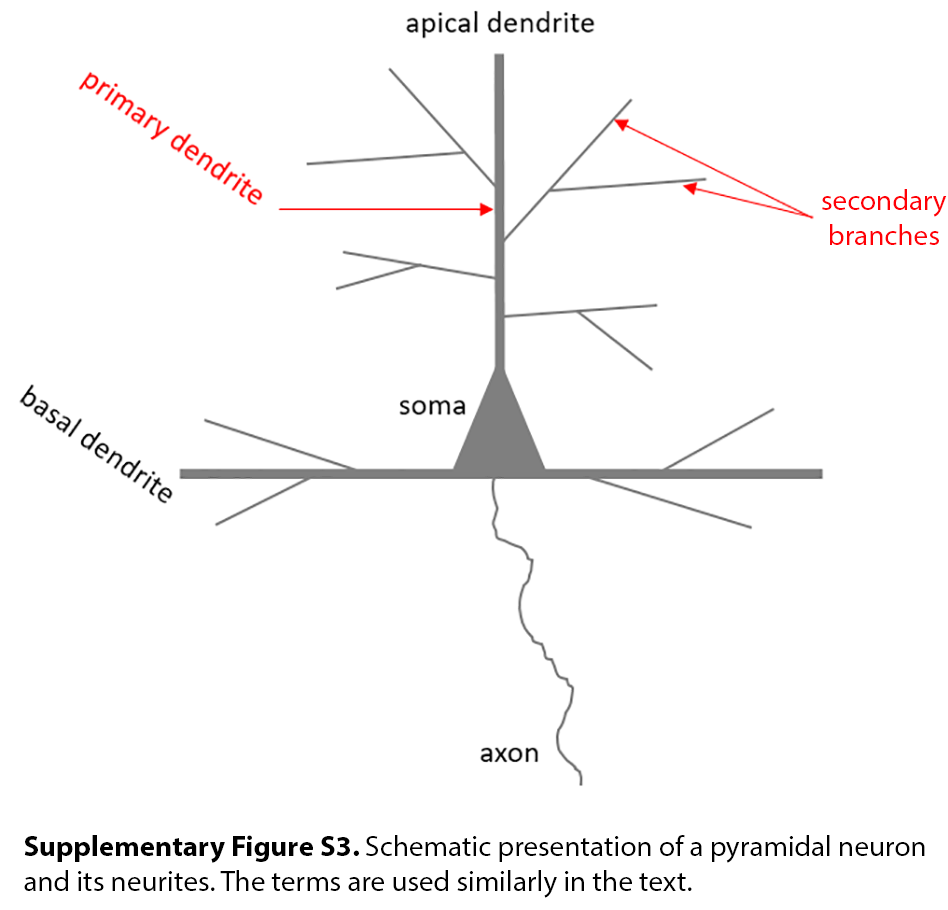

Supplement: Supplementary file 3 [file Image_3.TIF]

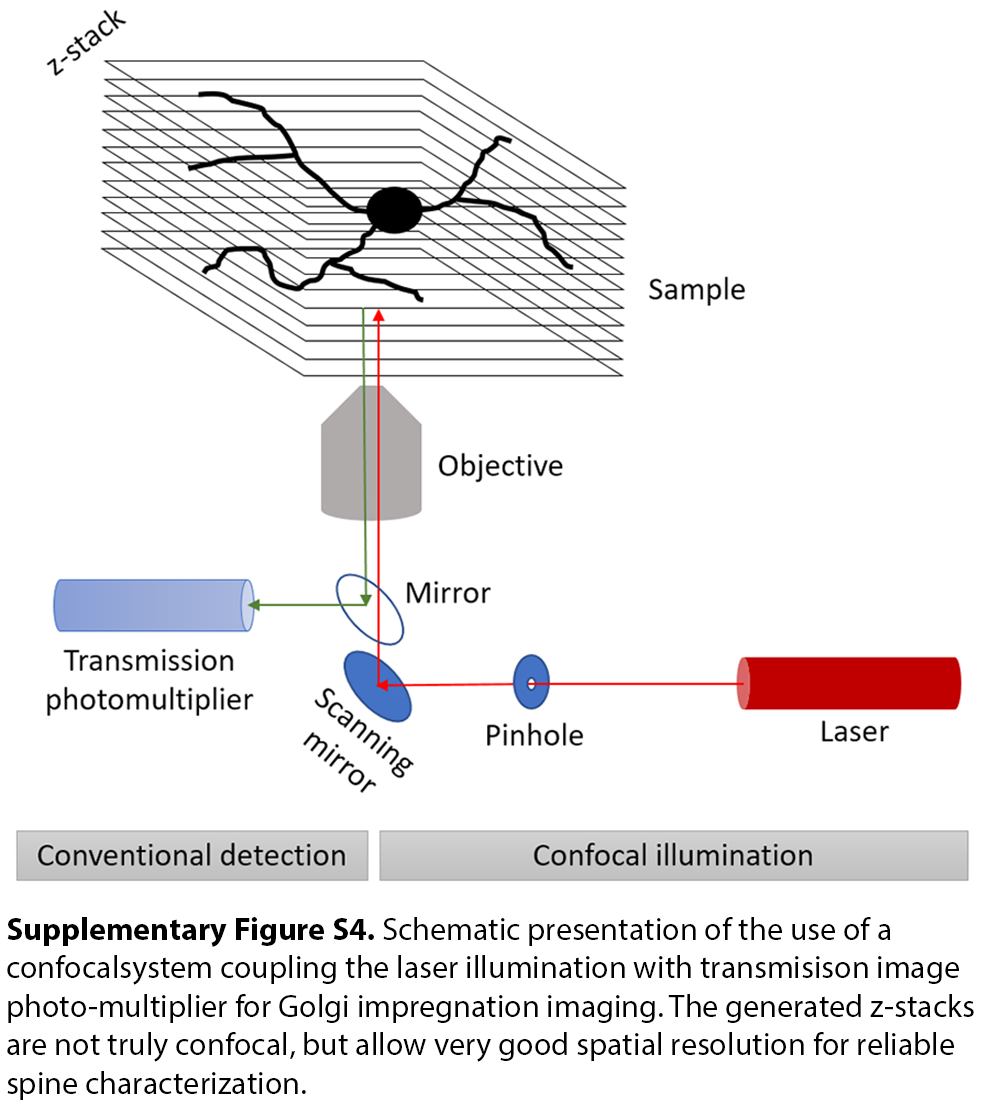

Supplement: Supplementary file 4 [file Image_4.TIF]
